# Linking timing to nitrogen use efficiency: Rice OsEC-Ghd7-ARE1 module works on it

**DOI:** 10.1093/plphys/kiae488

**Published:** 2024-09-12

**Authors:** Munkhtsetseg Tsednee

**Affiliations:** Plant Physiology, American Society of Plant Biologists; Agricultural Biotechnology Research Center, Academia Sinica, Taipei 11529, Taiwan

High-yielding cereal varieties, for example, semi-dwarf rice and wheat, developed during the Green Revolution in the 1960s transformed global agriculture and reduced poverty (Liu et al. 2022). However, the high requirements of these cereal varieties for essential nutrient nitrogen (N) increased its fertilizer inputs over 10 times since then. Current high consumption of N fertilizers exceeds 110 million tons per year worldwide (Hu et al. 2023) and, undesirably, also contributes to global environmental changes with serious threats because of excess N pollution.

Crop production in Asian countries, including rice (*Oryza sativa* L.) farming, uses nearly 50% of the total N fertilizers. Yet rice takes up only 30% to 50% of the N supplied (Hu et al. 2023). Therefore, improving N use efficiency (NUE) in rice is a major challenge for “producing more with less inputs.”

For NUE, the absorption from soil is a critical factor. Rice roots acquire N in the forms of ammonium (NH_4_^+^) and nitrate (NO_3_^−^). Although the efforts to improve their NUE in uptake by engineering N transporters have yielded benefits (Liu et al. 2022), new targets and approaches are still sought.

In this issue of *Plant Physiology*, Sun et al. (2024) surveyed N uptake rates in rice mutants and identified a flowering regulator gene involved in the modulation of ammonium uptake. The authors first generated rice mutants in Geng/*japonica* (GJ) variety using γ-irradiation and quantified ^15^NH_4_^+^ influx rates in a random selection of 100 mutant roots. They further found a mutant that has an enhanced N uptake ability, approximately double-fold of wild type, and less sensitivity to N supply. Using a map-based cloning, the authors then identified the *EARLY FLOWERING3-1* (*ELF3-1*) gene as its causal gene. To further confirm this gene function, the authors created *OsELF3-1* overexpression and CRISPR/Cas9 knockout mutant lines showing significant reductions and increases in their ^15^NH_4_^+^ uptake rates, respectively. These results verified that *OsELF3-1* acts as a negative regulator of ammonium uptake in rice.

Previously, *OsELF3-1* was known to regulate rice circadian system and flowering (Zhao et al. 2012). In *Arabidopsis*, it functions by forming the evening complex (EC) with ELF4 and LUX transcription factors (Nusinow et al. 2011). Therefore, to determine if the OsEC complex forms in rice, the authors performed yeast 2-hybrid and pull-down assays. They observed that OsELF3-1 physically interacts with 3 OsELF4s and OsLUX and, using bimolecular fluorescence complementation, showed that OsELF3-1 guides the OsEC complex to the nucleus.

Phenotypically, *oself3-1* mutants show delays in heading dates both under low and high N-supplied conditions and exhibit increased expression of *Grains Height Date-7* (*Ghd7*), a negative regulator of photoperiodic heading control (Sun et al. 2024).

In line with this observation, the identification of LUX binding sites, the LBS motif, in *Ghd7*'s promoter led the authors to hypothesize that *OsELF3-1* regulates *Ghd7* expression through the OsEC complex. Indeed, the results from their DNA–protein interaction assays showed that LUX binds to the *Ghd7*'s LBS motif, and the presence of OsELF3-1 and OsELF4s enhances this binding. The LUX binding to *Ghd7* further suppresses its expression, examined by a transcriptional activity assay, and the formation of OsLUX-OsELF3-1-OsELF4s complex enhances this suppression. Thus, these results suggest that the LUX transcription factor, in the OsEC complex form, binds directly to *Ghd7* to suppress its expression.

Furthermore, using CRISPR/Cas9-edited *ghd7* mutants that take up less ^15^NH_4_^+^, the authors confirmed the *Ghd7*'s positive regulation on N uptake. Consistent with a previous report (Wang et al. 2018), their transient expression assay results showed that *Ghd7* suppresses *ABC1 REPRESSOR1* (*ARE1*) expression, a negative regulator of NUE. Consequently, the *are1* mutants showed an improved N acquisition. Moreover, *oself3-1ghd7* double mutant behaved as a *ghd7* single mutant with reduced N uptake, suggesting that these gene products are on the same regulatory pathway and *Ghd7* is a downstream regulator.

Altogether, the results reveal that OsEFL3-1 forms a ternary complex, the OsEC, with OsELF4s and OsLUX, and guides the complex to the nucleus to further bind to *Ghd7* to suppress *Ghd7*'s expression. This suppression alters *Ghd7*'s inhibition to *ARE1*, resulting in reduced N uptake (Fig. A). However, in the *oself3-1* mutant, a disruption of the OsEC complex formation diminishes its suppression to *Ghd7*; in turn, it enhances N uptake due to strongly inhibited *ARE1* expression by *Ghd7* (Fig. B).

**Figure.**
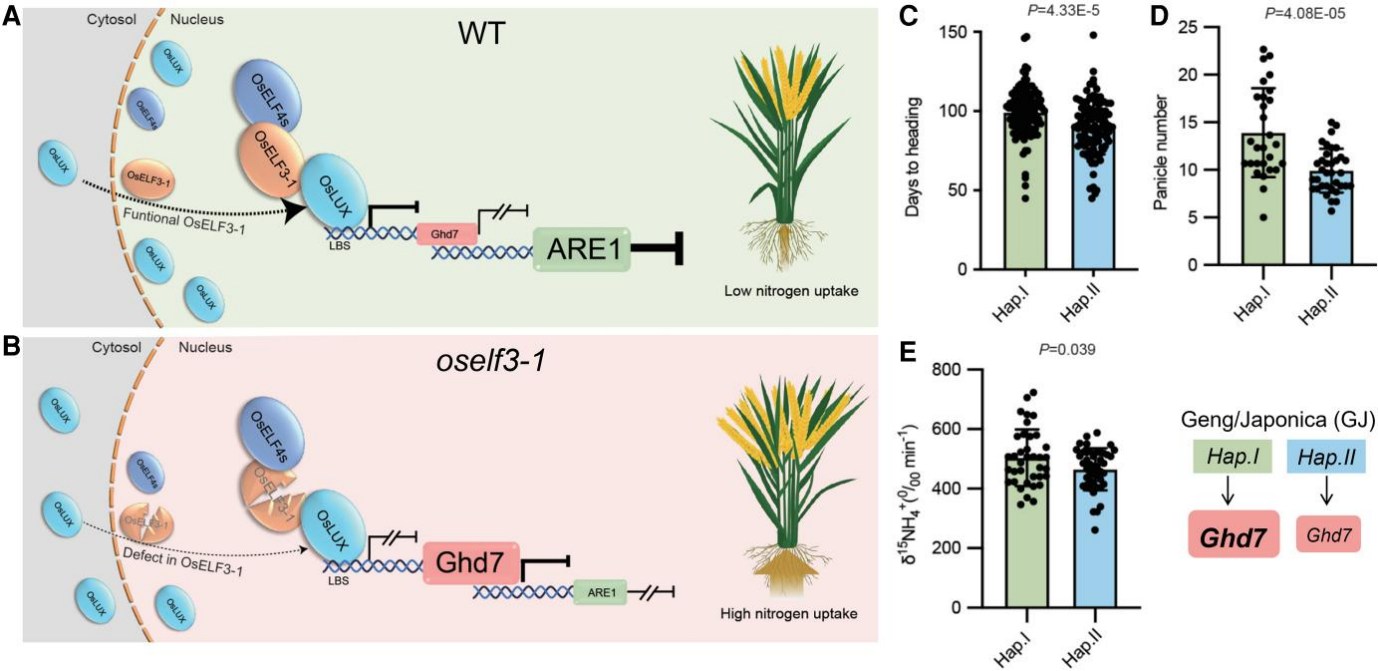
Proposed working model for the OsEC-Ghd7-ARE1 pathway in the regulation of nitrogen uptake and phenotypes of GJ haplotypes. **A)** In wild-type (WT), OsELF3-1 contributes to the translocation of OsLUX to the nucleus. The OsEC, comprising OsELF4s, OsELF3-1, and OsLUX, binds to the LBS motif in *Ghd7*'s promoter, suppressing its expression. This suppression reduces *Ghd7*'s inhibition of *ARE1*, leading to decreased N uptake in plants. **B)** Conversely, mutations in OsELF3-1 disrupt OsEC formation, diminishing its ability to suppress *Ghd7*. Elevated *Ghd7* expression then strongly represses *ARE1*, ultimately enhancing plant N uptake. **C to E)** The days to heading, panicle number, and ^15^NH_4_^+^ uptake ratio of 2 GJ haplotypes, Hap.I and Hap.II. The simplified diagram represents the *Ghd7* expression levels in the haplotypes (modified from Sun et al. (2024)).

Interestingly, *Ghd7-*regulated traits, including delayed heading date, increased panicle number, and enhanced ^15^NH_4_^+^ uptake, were observed in GJ haplotype Hap.I, in association with its elevated *Ghd7* expression level, compared with that of GJ haplotype Hap.II (Fig. C to E). Thus, the authors recommend *Hap.I* in breeding applications in mid-latitude regions.

In summary, Sun et al. (2024) report a new target, the OsEC-Ghd7-ARE1 module, and a breeding strategy, Hap.I type GJ variety, for further enhancement of N uptake and NUE in rice. Moreover, a new function of the flowering gene *OsELF3-1* in regulating N uptake expands our understanding of photoperiodic regulation to essential nutrient acquisition.

## Data Availability

No data associated with this article.
